# Syntheses, spectroscopy, and crystal structures of 3-(4-bromo­phen­yl)-1,5-di­phenyl­formazan and the 3-(4-bromo­phen­yl)-1,5-di­phenyl­verdazyl radical and the crystal structure of the by-product 5-anilino-3-(4-bromo­phen­yl)-1-phenyl-1*H*-1,2,4-triazole

**DOI:** 10.1107/S2056989018001913

**Published:** 2018-02-07

**Authors:** Gregor Schnakenburg, Andreas Meyer

**Affiliations:** a University of Bonn, Institute of Inorganic Chemistry, Gerhard-Domagk-Strasse 1, 53121 Bonn, Germany; b University of Bonn, Institute of Physical and Theoretical Chemistry, Wegelerstrasse 12, 53115 Bonn, Germany

**Keywords:** crystal structure, H-atom transfer, tautomerism, hydrogen bonding, radical, dye, heterocycle

## Abstract

The title compounds, C_19_H_15_BrN_4_, C_20_H_16_BrN_4_ and C_20_H_15_BrN_4_, are nitro­gen-rich organic compounds that are related by their synthesis. The verdazyl radical, in which stacking leads to anti­ferromagnetic inter­actions, was reported previously [Iwase *et al.* (2013 ▸). *Phys. Rev. B*, **88**, 184431]. For this compound, improved structural data and spectroscopic data are presented. The other two compounds have been crystallized for the first time and form stacks of dimers, roughly along the *a*-axis direction of the crystal. The formazan mol­ecule shows signs of rapid intra­molecular H-atom exchange typical for this class of compounds and spectroscopic data are provided in addition to the crystal structure. The triazole compound appears to be a side-product of the verdazyl synthesis.

## Abstract 2

The syntheses of a formazan and a verdazyl radical are reported along with their crystal structures, UV–Vis spectra, and the EPR spectrum of the radical. In addition, the isolation of a possible by-product was achieved.

## Chemical context   

Verdazyl radicals are a family of organic radicals first reported by Kuhn & Trischmann (1963 ▸) who emphasized their intense green color and their stability. These Kuhn-verdazyls require formazan precursors, which are intensely red in color and inter­esting in their own respect (Nineham, 1955 ▸; Scudiero *et al.* 1988 ▸). A few years after Kuhn’s discovery, syntheses leading to the orange 6-oxo- and 6-thioxoverdazyls were developed (Neugebauer & Fischer, 1980 ▸; Neugebauer *et al.*, 1988 ▸). As of late, verdazyls experience renewed inter­est, partially as a result of the improvements concerning their syntheses, enabling the introduction of a large variety of substitution patterns (Paré *et al.*, 2005 ▸; Bancerz *et al.*, 2012 ▸; Matuschek *et al.*, 2015 ▸; Le *et al.*, 2017 ▸). Such tailor-made radicals have possible applications as fundamental building blocks in mol­ecular magnets or in spintronic materials (Koivisto & Hicks, 2005 ▸; Train *et al.*, 2009 ▸; Ratera & Veciana, 2012 ▸). Verdazyls often avoid stacking, preventing the occurrence of strong magnetic inter­actions. However, some exceptions to this rule have been reported, where strong anti­ferromagnetic coupling occurs as a consequence (Koivisto *et al.*, 2006 ▸; Eusterwiemann *et al.*, 2017 ▸). With respect to applications in spintronics, tetra­thia­fulvalene-substituted verdazyl compounds represent inter­esting examples (Chahma *et al.*, 2006 ▸; Venneri *et al.*, 2015 ▸). Herein, the preparation and crystal structures of three mol­ecules involved in verdazyl synthesis are reported. 3-(4-Bromo­phen­yl)-1,5-di­phenyl­formazan, C_19_H_15_N_4_Br (**1**), was used as the educt to obtain the 3-(4-bromo­phen­yl)-1,5-di­phenyl­verdazyl radical C_20_H_16_N_4_Br (**2**). Additionally, 5-anilino-3-(4-bromo­phen­yl)-1-phenyl-1*H*-1,2,4-triazole, C_20_H_15_N_4_Br (**3**), could be crystallized, representing a possible side-product in verdazyl synthesis. The identification of such by-products might aid future efforts to further elucidate the so-far poorly understood mechanism of verdazyl formation. The crystal structures of all three mol­ecules could be obtained and are discussed in detail for **1** and **3**. The structure of **2** has already been discussed by Iwase *et al.* (2013 ▸) and a dataset with improved residuals is provided herein. In addition to the crystal structures, spectroscopic data for **1** and **2** are presented.
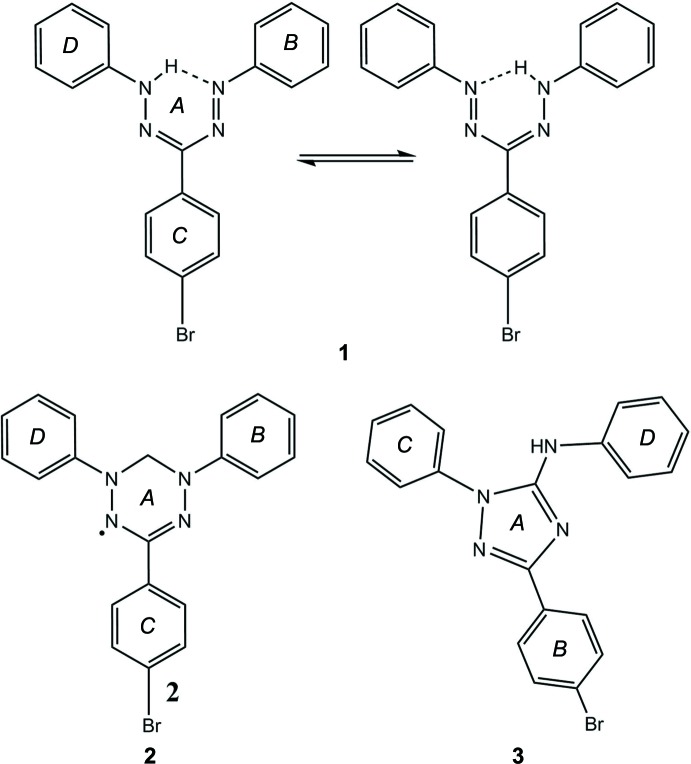


## Structural commentary   

**Figure 1:**
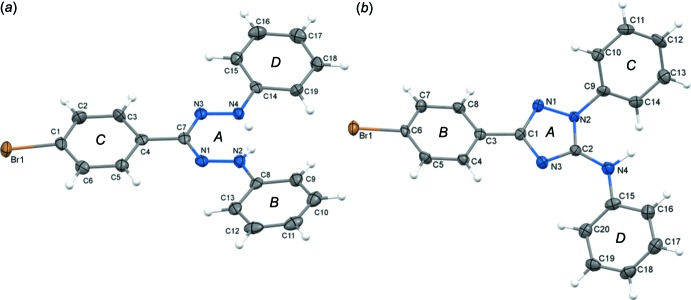
The mol­ecular structures of (*a*) **1** and (*b*) **3** with displacement ellipsoids drawn at the 50% probability level.

The mol­ecular structures of **1** and **3** are shown in Fig. 1 ▸ *a* and *b*, respectively. Compound **2** has a structure typical for verdazyls, for details see Iwase *et al.* (2013 ▸). For **1**, inter­esting structural features are the bond lengths in the central NNCNN atomic chain. Taking into account the 3σ criterion, the bond lengths N1—N2 and N3—N4 are identical [1.309 (5) and 1.300 (5) Å, respectively] and the same is true for N1—C7 and N3—C7 [1.350 (5) and 1.364 (5) Å, respectively]. These bond lengths lie between values typical for single and double bonds. The pairwisely identical bond lengths are in agreement with rapid intra­molecular H-atom exchange (Nineham, 1955 ▸; Otting & Neugebauer, 1969 ▸; Buemi *et al.*, 1998 ▸). Correspondingly, the H atom was considered to be split between the two possible positions at N2 and N4. In both positions, an intra­molecular hydrogen bond is formed with H⋯*A* distances amounting to 1.93 (10) Å for N2—H2⋯N4 and 1.86 (12) Å for N4—H4⋯N2 (Table 1 ▸). Finally, it is noted that the mol­ecule is essentially planar with angles between the normal vectors of the NNCNN mean plane *A* and the three rings *B*, *C*, and *D* amounting to 9.71 (16) (*A*/*B*), 5.28 (3) (*A*/*C*), and 12.18 (13)° (*A*/*D*).

Compound **3** was isolated in later fractions of the column that was used to purify **2**. Such triazole compounds have been identified as products of thermal verdazyl decomposition at 473 K or after four days of refluxing at 353 K in benzene (Neugebauer *et al.*, 1972 ▸). Here, the formation of **3** was observed under much less harsh conditions. The bond lengths within ring *A* suggest bond orders between single and double bonds, in accordance with the aromatic character of 1,2,4-triazoles. Closer inspection reveals that three of the five bonds are considerably longer than the other two [N1—N2 = 1.375 (4), N2—C2 = 1.358 (5), and N3—C1: 1.370 (5) Å compared to N1—C1 = 1.321 (5) and N3—C2 = 1.326 (5) Å], indicating that the resonance structure given in Fig. 5 ▸ is the most important one. The amino-nitro­gen N4 is connected to ring *A* by a bond of similar character to the bonds within the ring [N4—C2 = 1.371 (5) Å] whereas its bond to phenyl ring *D* has essentially single-bond character [N4—C15 = 1.426 (6) Å]. The bonds connecting ring *A* with rings *B* and *C* also have mostly single-bond character [C1—C3 = 1.479 (5) and N2—C9 = 1.441 (5) Å). The mean planes of rings *B*, *C*, and *D* are tilted with respect to the mean plane of *A* and are arranged in a propeller-like manner [angles between normal vectors: *A*/*B =* 14.47 (14), *A*/*C =* 40.42 (14), and *A*/*D =* 20.67 (16)°].

## Supra­molecular features   

**Figure 2:**
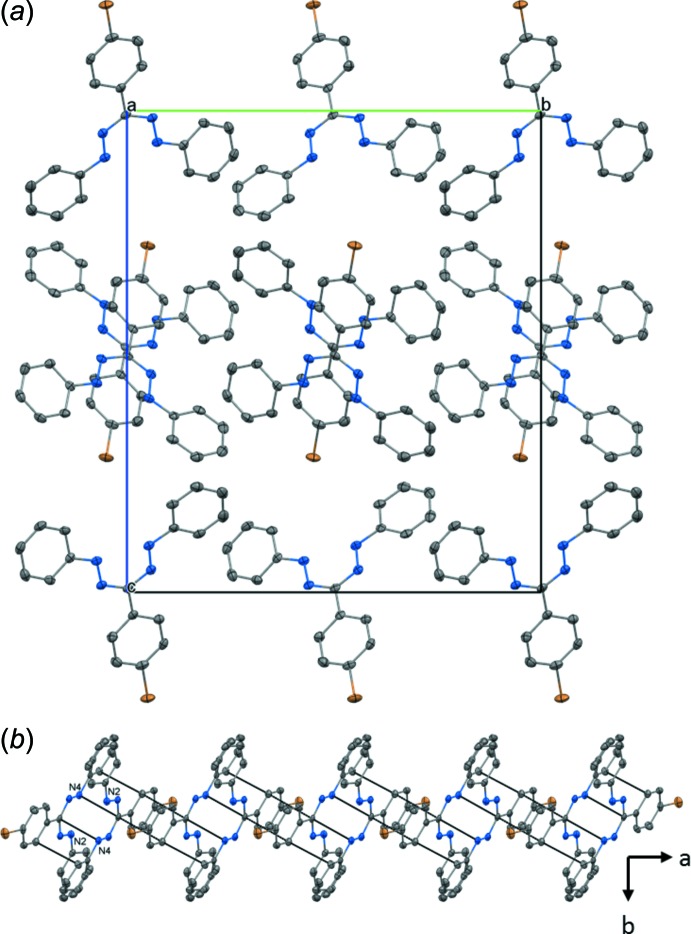
(*a*) Unit cell of **1** viewed parallel to the (100) plane. (*b*) Stacks of dimers formed along the *a*-axis direction. Two nitro­gen atoms of two mol­ecules are labelled.

Compound **1** crystallizes with ortho­rhom­bic symmetry in space group *Pbca*, in which head-to-tail dimers between two mol­ecules are stacked along the *a*-axis direction (Fig. 2 ▸). Within a dimer, the shortest contacts are 3.213 (5) and 3.372 (6) Å for N4⋯C7 and C19⋯C5, respectively. The short C5⋯C7 contact [3.277 (6) Å] connects pairs of dimers. The Br atom is not involved in halogen bonding, which is a structural motive attracting increasing attention (Metrangolo *et al.*, 2008 ▸; Gilday *et al.*, 2015 ▸). Relatively short contacts between H19 as well as H9 and the Br1 atom of another mol­ecule connect different stacks (Table 1 ▸). However, the observed distances of 3.05 Å (C19—H19⋯Br1) and 3.14 Å (C9—H9⋯Br1) are still longer than the sum of the van der Waals radii of H and Br, meaning that these are at best very weak hydrogen bonds.

The packing of **2** leading to anti­ferromagnetic coupling has already been described (Iwase *et al.*, 2013 ▸).

**Figure 3:**
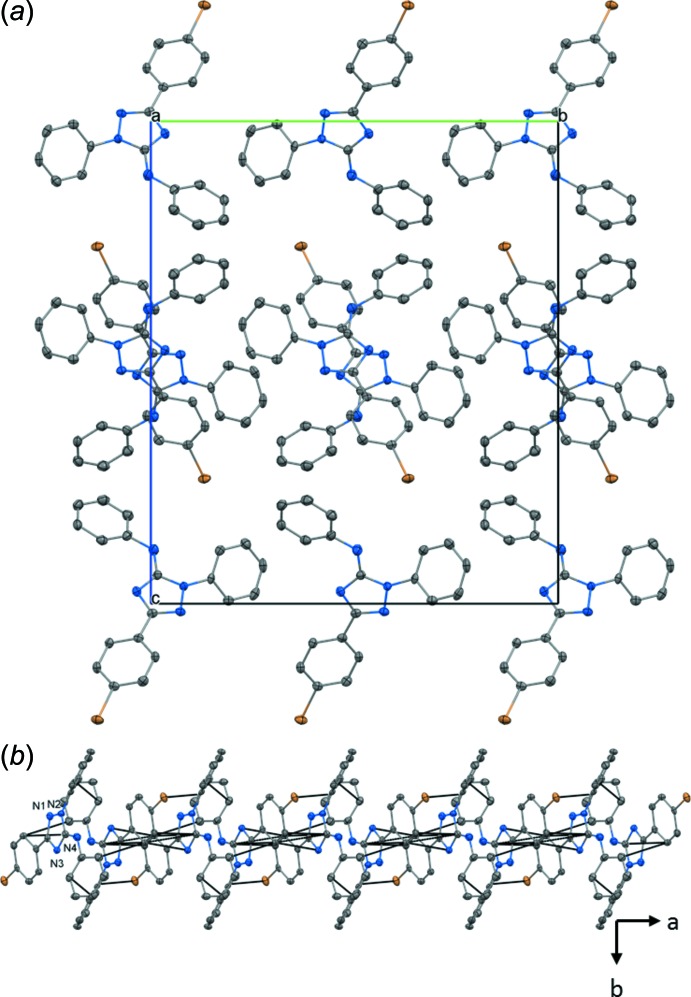
(*a*) Unit cell of **3** viewed parallel to the (100) plane. (*b*) Stacks of dimers formed along the *a*-axis direction. The nitro­gen atoms of one mol­ecule are labelled.

Compound **3** has a similar structure to **1** in space group *Pbca* and with pairs of mol­ecules stacked along the *a*-axis direction (Fig. 3 ▸). Here, the centroid-to-centroid distances of the *A* rings are 3.564 (3) and 4.661 (3) Å within and between the dimers, respectively. However, the shortest intra-dimer contact is a C—H⋯π inter­action (Table 2 ▸) between rings *C* and *D* (C10—H10⋯C20, 2.75 Å). A similar contact is found between H17 and C19 (C17—H17⋯C19, 2.84 Å), forming a contact between different stacks. π-Stacking is observed between rings *A* and *B*, connecting pairs of dimers, with the shortest contacts being 3.229 (6) (C8⋯N3), 3.318 (6) (C8⋯C2), and 3.378 (6) Å (C7⋯C2). As with **1** and **2**, no halogen bonding is observed, but the Br atom is involved in a very weak hydrogen bond (C14—H14⋯Br1, 2.99 Å; Table 2 ▸).

## Spectroscopy   

**Figure 4:**
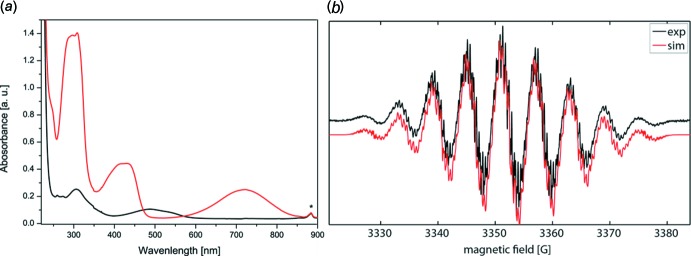
(*a*) UV–Vis spectra of **1** (black line, 2.3 µ*M*, DCM) and **2** (red line, 11 µ*M*, DCM). (*b*) EPR spectrum of **2** in degassed deuterated DCM (black line) along with its simulation (red line) obtained using the program *EasySpin* (Stoll & Schweiger, 2006 ▸).

Fig. 4 ▸ *a* shows the UV–Vis spectra of **1** and **2**, while Fig. 4 ▸ *b* represents the EPR spectrum of **2** and its simulation (black and red lines, respectively). The UV–Vis spectra of **1** and **2** are typical for formazans and verdazyls, respectively, with the peaks in the visible range at 490 nm (**1**) as well as at 425 and 720 nm (**2**) being responsible for their intense red (**1**) or green colors (**2**). The EPR spectrum of **2** was simulated by assuming a *g* value of 2.00354 and hyperfine coupling constants (HFCC) of 16.77 and 16.48 MHz for the two pairs of nitro­gen nuclei. In addition, the approximate values for the HFCC of the phenyl ring protons could be obtained, amounting to 0.01 (CH_2_), 3.04 (H, rings *B* and *D*, *ortho*), 1.14 (H, rings *B* and *D*, *meta*), 3.34 (H, rings *B* and *D*, *para*), 1.14, (H, ring *C*, *ortho*), and 0.52 MHz (H, ring *C*, *meta*). The assignment of the protons is in accordance with that of Kopf *et al.* (1971 ▸).

## Database survey   

The Cambridge Structural Database (CSD, Version 5.36; Groom *et al.*, 2016 ▸) was queried for other formazans, verdazyls, and 1,2,4-triazoles. The search revealed 21 examples of formazans if the only restriction was to have carbon substit­uents in the 1,3,5-positions. This number reduced to nine if all of these substituents were required to be phenyl-based, one of these nine examples being a metal complex of a formazan. The remaining eight structures include examples in which the bond lengths in the NNCNN unit alternate, as well as examples in which they are pairwisely equal in a similar manner to that described herein. Inter­estingly, 3,5-diphenyl-1-(4-bromo­phen­yl)formazan (regioisomer of **1**, CCDC code EMEVUO; Tunç & Yıldırım, 2010 ▸) shows alternating bond lengths, which reflects the fact that the two nitro­gen atoms are chemically inequivalent in this mol­ecule. An example with split hydrogen positions is 1,5-diphenyl-3-(*p*-nitro­phen­yl)formazan (GUHCIW; Iqbal *et al.*, 2009 ▸), which shows a similar stacking to that observed in **1** and can be formally derived from **1** by replacing the bromine with a nitro group. 33 examples for 1,3,5-aryl-substituted verdazyls were found in the CSD, only 14 of them Kuhn-verdazyls. The largest hitlist was obtained for 1,3,5-substituted 1,2,4-triazoles (1001 entries). This number reduced drastically if purely organic compounds were considered exclusively (42 hits) and even further if the substitutent at C5 was required to be a nitro­gen atom (four hits, no further restriction).

## Synthesis and crystallization   

**Figure 5:**
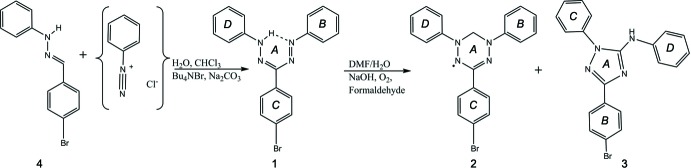
Synthesis of **1**, **2**, and **3**.

The syntheses were performed following Berry *et al.*, 2009 ▸ (Fig. 5 ▸). The hydrazone **4** required for the synthesis of **1** was synthesized by refluxing a solution of *p*-bromo­benzaldehyde with phenyl­hydrazine in ethanol and collecting the slightly yellow precipitate that formed after cooling the solution down to room temperature (rt).

To synthesize **1**, 450 mg (1.72 mmol) of **4** and 80 mg (0.25 mmol) of tetra­butyl­ammonium bromide were dissolved in 11 mL of di­chloro­methane (DCM) and combined with 1.4 g of sodium carbonate in 11 mL of water to form a biphasic system, which was stirred at 273 K for 30 min. During this time, 1.8 mL (186 mg, 2 mmol) of aniline were dissolved in 4.5 mL of dilute hydro­chloric acid (*ca* 12%) and stirred at 273 K. To this solution, 55 mg (3.3 mmol) of sodium nitrite in 3 mL of water were added dropwise over the course of ten minutes, leading to the occurrence of a slight yellow color. This yellow solution was added carefully to the biphasic solution of **4** and an intense red color evolved within minutes. After one h, 20 mL of water were added and the temperature was allowed to increase to rt. After stirring for another 30 minutes at rt, the phases were separated. The organic phase was washed with water and dried over Na_2_SO_4_ before removing the solvent under reduced pressure. The raw product was subjected to column chromatography using aluminum oxide (AlOx, water content 5%) as stationary phase and DCM/cyclo­hexane (1:4). The red fractions were collected, yielding **1** as red solid in 66% yield (307 mg). Crystals of **1** were obtained by dissolving the solid in a mixture of DCM and hexane which was left to evaporate.

To obtain **2**, 119 mg (0.31 mmol) of **1** were dissolved in 10 mL of di­methyl­formamide and mixed with 0.7 mL 2 *M* aqueous sodium hydroxide solution and 0.65 mL of 37% formaldehyde solution. The mixture was stirred at rt in an open vessel with contact to air, leading to a change of color from red to green over the course of an hour. 20 mL of water and diethyl ether were then added to the solution and the phases were separated from each other. After drying the organic phase over Na_2_SO_4_, the raw product was subjected to column chromatography using AlOx (water content 5%) and di­ethyl­ether/cyclo­hexane (1:5) as eluent. The green fractions were collected and the solvent was removed under reduced pressure (yield: 37 mg, 30%). Crystals of **2** were obtained by dissolving the product in a mixture of DCM and hexane and leaving the green solution to evaporate.

Compound **3** was obtained by collecting the slightly yellow fractions that eluted from the column after **2** and removing the solvent. Dissolving the resulting brownish solid in a mixture of DCM and hexane and leaving the solution to evaporate afforded crystals suitable for X-ray crystallography.

**Additional analytical data for 1 and 2. 1**: ^1^H NMR (400 MHz, DCM-*d*_2_): δ 15.45 (*s*, 1H); 8.08 (*dt*, *J* = 8.8 MHz, 2.2 MHz, 2H); 7.75 (*dm*, *J* = 8.4 MHz, 4H); 7.61 (*dt*, *J* = 8.8 MHz, 2.2 MHz, 2H); 7.52 (*ddt*, *J* = 8.4 MHz, 7.2 MHz, 1.6 MHz, 4H); 7.36 (*tt*, *J* = 7.2 MHz, 1.2 MHz, 2H). ESI–MS (positive, *m*/*z*): calculated 377.04 ([*M* − H]^+^), found 377.04. UV–Vis: see above.

**2**: ESI–MS (positive, *m*/*z*): calculated 391.06 ([*M*] ^+^), found 391.06. UV–Vis and EPR: see above.

## Refinement   

Crystal data, data collection and structure refinement details are summarized in Table 3 ▸. C-bound H atoms were refined using a riding model with C—H = 0.95–0.99 Å and *U*_iso_(H) = 1.2*U*_eq_(C). N-bound H atoms were located in a difference-Fourier map and refined with *U*_iso_(H) = 1.2*U*_eq_(N).

## Supplementary Material

Crystal structure: contains datablock(s) global, 1, 2, 3. DOI: 10.1107/S2056989018001913/lh5869sup1.cif

Structure factors: contains datablock(s) 1. DOI: 10.1107/S2056989018001913/lh58691sup2.hkl

Supporting information file. DOI: 10.1107/S2056989018001913/lh58691sup5.cdx

Supporting information file. DOI: 10.1107/S2056989018001913/lh58691sup6.cml

Structure factors: contains datablock(s) 2. DOI: 10.1107/S2056989018001913/lh58692sup3.hkl

Supporting information file. DOI: 10.1107/S2056989018001913/lh58692sup7.cml

Structure factors: contains datablock(s) 3. DOI: 10.1107/S2056989018001913/lh58693sup4.hkl

Supporting information file. DOI: 10.1107/S2056989018001913/lh58693sup8.cml

CCDC references: 1821241, 1821240, 1821239

Additional supporting information:  crystallographic information; 3D view; checkCIF report

## Figures and Tables

**Table 1:** Hydrogen-bond geometry (Å, °) for **1** [Chem scheme1]

*D*—H⋯*A*	*D*—H	H⋯*A*	*D*⋯*A*	*D*—H⋯*A*
N2—H2⋯N4	0.80 (10)	1.93 (10)	2.566 (5)	137 (9)
N4—H4⋯N1	0.81 (12)	2.40 (11)	2.803 (5)	112 (9)
N4—H4⋯N2	0.81 (12)	1.86 (12)	2.566 (5)	145 (10)
C19—H19⋯Br1^i^	0.95	3.05	3.921 (4)	153
C9—H9⋯Br1^i^	0.95	3.14	4.014 (5)	153

Symmetry code: (i) 
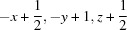
.

**Table 2:** Hydrogen-bond geometry (Å, °) for **3** [Chem scheme1]

*D*—H⋯*A*	*D*—H	H⋯*A*	*D*⋯*A*	*D*—H⋯*A*
C14—H14⋯Br1^i^	0.95	2.99	3.814 (4)	146 (1)
C10—H10⋯C20^ii^	0.95	2.75	3.575 (5)	146 (1)
C17—H17⋯C19^iii^	0.95	2.84	3.694 (6)	150 (1)

Symmetry codes: (i) 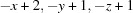; (ii) 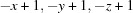; (iii) 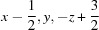.

**Table 3:** Experimental details

	**1**	**2**	**3**
Crystal data			
Chemical formula	C_19_H_15_BrN_4_	C_20_H_16_BrN_4_	C_20_H_15_BrN_4_
*M*_r_	379.26	392.28	391.27
Crystal system, space group	Orthorhombic, *P* *b* *c* *a*	Orthorhombic, *P* *b* *c* *a*	Orthorhombic, *P* *b* *c* *a*
Temperature (K)	100	123	100
*a*, *b*, *c* (Å)	7.7930 (5), 19.0947 (16), 22.1843 (17)	7.0881 (3), 21.2183 (11), 22.2028 (9)	7.7989 (9), 18.971 (3), 22.455 (4)
*V* (Å^3^)	3301.1 (4)	3339.2 (3)	3322.4 (8)
*Z*	8	8	8
Radiation type	Mo *K*α	Mo *K*α	Mo *K*α
μ (mm^−1^)	2.50	2.47	2.48
Crystal size (mm)	0.33 × 0.06 × 0.04	0.15 × 0.12 × 0.06	0.32 × 0.16 × 0.1

Data collection			
Diffractometer	Bruker D8 Venture	Stoe IPDS 2T	Bruker X8 Kappa APEXII
Absorption correction	Multi-scan (*SADABS*; Bruker, 2015 ▸)	Integration (*X-RED32*; Stoe & Cie, 2009 ▸)	Multi-scan (*SADABS*; Bruker, 2015 ▸)
*T*_min_, *T*_max_	0.550, 0.746	0.254, 0.620	0.583, 0.746
No. of measured, independent and observed [*I* > 2σ(*I*)] reflections	60778, 3975, 2652	70879, 3640, 3397	19450, 3999, 2708
*R*_int_	0.147	0.128	0.098
(sin θ/λ)_max_ (Å^−1^)	0.661	0.639	0.660

Refinement			
*R*[*F* ^2^ > 2σ(*F* ^2^)], *wR*(*F* ^2^), *S*	0.057, 0.153, 1.03	0.029, 0.076, 1.10	0.056, 0.126, 1.05
No. of reflections	3975	3640	3999
No. of parameters	224	226	229
H-atom treatment	H atoms treated by a mixture of independent and constrained refinement	H-atom parameters constrained	H atoms treated by a mixture of independent and constrained refinement
Δρ_max_, Δρ_min_ (e Å^−3^)	1.76, −1.08	0.72, −0.65	1.46, −0.84

Computer programs: *APEX2* and *SAINT* (Bruker, 2015 ▸), *X-AREA* (Stoe & Cie, 2009 ▸), *SHELXS97* and *SHELXL97* (Sheldrick, 2008 ▸) and *OLEX2* (Dolomanov *et al.*, 2009 ▸).

## supplementary crystallographic information

### N'-Anilino-4-bromo-N-(phenylimino)benzene-1-carboximidamide (1) Crystal data

**Table:**

C_19_H_15_BrN_4_	*D*_x_ = 1.526 Mg m^−^^3^
*M**_r_* = 379.26	Mo *K*α radiation, λ = 0.71073 Å
Orthorhombic, *P**b**c**a*	Cell parameters from 9908 reflections
*a* = 7.7930 (5) Å	θ = 3.0–27.7°
*b* = 19.0947 (16) Å	µ = 2.50 mm^−^^1^
*c* = 22.1843 (17) Å	*T* = 100 K
*V* = 3301.1 (4) Å^3^	Needle, clear red
*Z* = 8	0.33 × 0.06 × 0.04 mm
*F*(000) = 1536	

### N'-Anilino-4-bromo-N-(phenylimino)benzene-1-carboximidamide (1) Data collection

**Table:**

Bruker D8 Venture diffractometer	3975 independent reflections
Radiation source: microfocus sealed X-ray tube, Incoatec Iµs	2652 reflections with *I* > 2σ(*I*)
Mirror optics monochromator	*R*_int_ = 0.147
Detector resolution: 7.9 pixels mm^-1^	θ_max_ = 28.0°, θ_min_ = 2.3°
ω and φ scans	*h* = −10→10
Absorption correction: multi-scan (SADABS; Bruker, 2015)	*k* = −25→25
*T*_min_ = 0.550, *T*_max_ = 0.746	*l* = −28→29
60778 measured reflections	

### N'-Anilino-4-bromo-N-(phenylimino)benzene-1-carboximidamide (1) Refinement

**Table:**

Refinement on *F*^2^	0 restraints
Least-squares matrix: full	Hydrogen site location: mixed
*R*[*F*^2^ > 2σ(*F*^2^)] = 0.057	H atoms treated by a mixture of independent and constrained refinement
*wR*(*F*^2^) = 0.153	*w* = 1/[σ^2^(*F*_o_^2^) + (0.0596*P*)^2^ + 12.5159*P*] where *P* = (*F*_o_^2^ + 2*F*_c_^2^)/3
*S* = 1.03	(Δ/σ)_max_ = 0.001
3975 reflections	Δρ_max_ = 1.76 e Å^−^^3^
224 parameters	Δρ_min_ = −1.08 e Å^−^^3^

### N'-Anilino-4-bromo-N-(phenylimino)benzene-1-carboximidamide (1) Special details

**Table:**

Geometry. All esds (except the esd in the dihedral angle between two l.s. planes) are estimated using the full covariance matrix. The cell esds are taken into account individually in the estimation of esds in distances, angles and torsion angles; correlations between esds in cell parameters are only used when they are defined by crystal symmetry. An approximate (isotropic) treatment of cell esds is used for estimating esds involving l.s. planes.

### N'-Anilino-4-bromo-N-(phenylimino)benzene-1-carboximidamide (1) Fractional atomic coordinates and isotropic or equivalent isotropic displacement parameters (Å^2^)

**Table:**

	*x*	*y*	*z*	*U*_iso_*/*U*_eq_	Occ. (<1)
Br1	−0.16420 (6)	0.55022 (3)	0.27999 (2)	0.03107 (17)	
N1	0.2728 (4)	0.55820 (18)	0.54659 (16)	0.0192 (7)	
N2	0.3581 (4)	0.5531 (2)	0.59722 (16)	0.0203 (8)	
H2	0.400 (12)	0.516 (6)	0.605 (4)	0.024*	0.54 (7)
N3	0.3438 (4)	0.43883 (18)	0.51495 (15)	0.0192 (7)	
N4	0.4238 (4)	0.4268 (2)	0.56531 (16)	0.0193 (8)	
H4	0.431 (14)	0.461 (6)	0.587 (5)	0.023*	0.46 (7)
C1	−0.0210 (5)	0.5366 (2)	0.34853 (19)	0.0240 (10)	
C2	0.0685 (6)	0.4745 (3)	0.3551 (2)	0.0278 (10)	
H2A	0.0650	0.4396	0.3247	0.033*	
C3	0.1633 (5)	0.4645 (2)	0.4072 (2)	0.0246 (9)	
H3	0.2257	0.4221	0.4120	0.029*	
C4	0.1701 (5)	0.5148 (2)	0.45276 (18)	0.0186 (8)	
C5	0.0796 (5)	0.5773 (2)	0.44382 (19)	0.0223 (9)	
H5	0.0836	0.6127	0.4738	0.027*	
C6	−0.0146 (5)	0.5881 (2)	0.39252 (19)	0.0228 (9)	
H6	−0.0753	0.6308	0.3871	0.027*	
C7	0.2672 (5)	0.5027 (2)	0.50893 (18)	0.0183 (8)	
C8	0.3693 (5)	0.6134 (2)	0.63337 (18)	0.0191 (9)	
C9	0.4789 (5)	0.6102 (2)	0.68286 (19)	0.0247 (9)	
H9	0.5375	0.5679	0.6920	0.030*	
C10	0.5026 (6)	0.6688 (3)	0.7187 (2)	0.0294 (10)	
H10	0.5793	0.6668	0.7520	0.035*	
C11	0.4152 (6)	0.7304 (3)	0.7065 (2)	0.0304 (11)	
H11	0.4326	0.7706	0.7308	0.036*	
C12	0.3018 (6)	0.7325 (2)	0.6580 (2)	0.0298 (11)	
H12	0.2400	0.7744	0.6499	0.036*	
C13	0.2776 (5)	0.6748 (2)	0.62164 (19)	0.0250 (10)	
H13	0.1993	0.6768	0.5889	0.030*	
C14	0.5102 (5)	0.3628 (2)	0.57135 (18)	0.0182 (8)	
C15	0.5307 (5)	0.3138 (2)	0.52541 (19)	0.0221 (9)	
H15	0.4798	0.3215	0.4871	0.027*	
C16	0.6253 (6)	0.2540 (2)	0.5358 (2)	0.0298 (10)	
H16	0.6399	0.2205	0.5045	0.036*	
C17	0.6999 (6)	0.2424 (2)	0.5923 (2)	0.0293 (10)	
H17	0.7645	0.2011	0.5993	0.035*	
C18	0.6798 (6)	0.2907 (2)	0.6376 (2)	0.0280 (10)	
H18	0.7328	0.2832	0.6756	0.034*	
C19	0.5834 (5)	0.3500 (2)	0.62810 (19)	0.0219 (9)	
H19	0.5663	0.3824	0.6600	0.026*	

### N'-Anilino-4-bromo-N-(phenylimino)benzene-1-carboximidamide (1) Atomic displacement parameters (Å^2^)

**Table:**

	*U*^11^	*U*^22^	*U*^33^	*U*^12^	*U*^13^	*U*^23^
Br1	0.0285 (2)	0.0471 (3)	0.0176 (2)	0.0039 (2)	−0.00492 (18)	0.0059 (2)
N1	0.0146 (15)	0.0221 (19)	0.0208 (18)	−0.0026 (14)	0.0021 (14)	0.0024 (15)
N2	0.0178 (17)	0.025 (2)	0.0176 (18)	−0.0008 (15)	−0.0007 (13)	0.0045 (16)
N3	0.0152 (16)	0.0239 (19)	0.0184 (17)	−0.0030 (14)	0.0024 (13)	0.0039 (14)
N4	0.0148 (16)	0.025 (2)	0.0175 (18)	−0.0009 (14)	−0.0008 (14)	0.0029 (15)
C1	0.0139 (19)	0.039 (3)	0.019 (2)	−0.0017 (18)	0.0004 (16)	0.0078 (19)
C2	0.030 (2)	0.029 (2)	0.025 (2)	0.004 (2)	0.0002 (18)	−0.006 (2)
C3	0.022 (2)	0.026 (2)	0.025 (2)	0.0079 (19)	−0.0024 (18)	0.0022 (18)
C4	0.0147 (17)	0.022 (2)	0.019 (2)	−0.0035 (17)	0.0046 (16)	0.0046 (17)
C5	0.020 (2)	0.025 (2)	0.021 (2)	−0.0013 (17)	0.0008 (17)	0.0008 (18)
C6	0.021 (2)	0.020 (2)	0.027 (2)	0.0000 (17)	0.0031 (18)	0.0077 (18)
C7	0.0126 (17)	0.024 (2)	0.018 (2)	−0.0054 (16)	0.0023 (15)	0.0052 (17)
C8	0.0183 (19)	0.023 (2)	0.016 (2)	−0.0047 (16)	0.0054 (15)	0.0018 (17)
C9	0.022 (2)	0.033 (3)	0.019 (2)	−0.0018 (19)	0.0015 (17)	0.0047 (18)
C10	0.026 (2)	0.039 (3)	0.023 (2)	−0.010 (2)	0.0032 (19)	−0.001 (2)
C11	0.034 (2)	0.028 (3)	0.030 (3)	−0.011 (2)	0.014 (2)	−0.004 (2)
C12	0.031 (2)	0.025 (2)	0.034 (3)	0.0007 (19)	0.017 (2)	0.004 (2)
C13	0.021 (2)	0.035 (3)	0.019 (2)	−0.0011 (19)	0.0046 (17)	0.0080 (19)
C14	0.0113 (17)	0.021 (2)	0.022 (2)	−0.0040 (16)	0.0011 (16)	0.0049 (17)
C15	0.020 (2)	0.027 (2)	0.019 (2)	−0.0057 (17)	0.0001 (17)	−0.0017 (18)
C16	0.031 (2)	0.022 (2)	0.036 (3)	−0.0025 (19)	0.006 (2)	−0.007 (2)
C17	0.029 (2)	0.022 (2)	0.037 (3)	0.0026 (19)	0.006 (2)	0.010 (2)
C18	0.027 (2)	0.034 (3)	0.023 (2)	0.006 (2)	0.0010 (19)	0.011 (2)
C19	0.022 (2)	0.027 (2)	0.017 (2)	0.0022 (18)	0.0035 (16)	−0.0002 (18)

### N'-Anilino-4-bromo-N-(phenylimino)benzene-1-carboximidamide (1) Geometric parameters (Å, º)

**Table:**

Br1—C1	1.904 (4)	C8—C13	1.398 (6)
N1—N2	1.309 (5)	C9—H9	0.9500
N1—C7	1.350 (5)	C9—C10	1.385 (7)
N2—H2	0.80 (10)	C10—H10	0.9500
N2—C8	1.405 (6)	C10—C11	1.385 (7)
N3—N4	1.300 (5)	C11—H11	0.9500
N3—C7	1.364 (5)	C11—C12	1.392 (7)
N4—H4	0.81 (12)	C12—H12	0.9500
N4—C14	1.401 (5)	C12—C13	1.379 (7)
C1—C2	1.384 (6)	C13—H13	0.9500
C1—C6	1.386 (6)	C14—C15	1.393 (6)
C2—H2A	0.9500	C14—C19	1.404 (6)
C2—C3	1.384 (6)	C15—H15	0.9500
C3—H3	0.9500	C15—C16	1.379 (6)
C3—C4	1.395 (6)	C16—H16	0.9500
C4—C5	1.400 (6)	C16—C17	1.398 (7)
C4—C7	1.476 (6)	C17—H17	0.9500
C5—H5	0.9500	C17—C18	1.373 (7)
C5—C6	1.370 (6)	C18—H18	0.9500
C6—H6	0.9500	C18—C19	1.375 (6)
C8—C9	1.392 (6)	C19—H19	0.9500
			
N2—N1—C7	119.3 (4)	C10—C9—C8	120.0 (4)
N1—N2—H2	117 (7)	C10—C9—H9	120.0
N1—N2—C8	117.5 (4)	C9—C10—H10	119.8
C8—N2—H2	126 (7)	C11—C10—C9	120.5 (4)
N4—N3—C7	116.9 (4)	C11—C10—H10	119.8
N3—N4—H4	113 (8)	C10—C11—H11	120.4
N3—N4—C14	117.8 (4)	C10—C11—C12	119.3 (4)
C14—N4—H4	128 (8)	C12—C11—H11	120.4
C2—C1—Br1	119.8 (3)	C11—C12—H12	119.5
C2—C1—C6	121.0 (4)	C13—C12—C11	121.0 (4)
C6—C1—Br1	119.1 (3)	C13—C12—H12	119.5
C1—C2—H2A	120.8	C8—C13—H13	120.3
C1—C2—C3	118.4 (4)	C12—C13—C8	119.5 (4)
C3—C2—H2A	120.8	C12—C13—H13	120.3
C2—C3—H3	119.0	N4—C14—C19	115.6 (4)
C2—C3—C4	122.0 (4)	C15—C14—N4	124.8 (4)
C4—C3—H3	119.0	C15—C14—C19	119.6 (4)
C3—C4—C5	117.7 (4)	C14—C15—H15	120.2
C3—C4—C7	121.6 (4)	C16—C15—C14	119.7 (4)
C5—C4—C7	120.7 (4)	C16—C15—H15	120.2
C4—C5—H5	119.5	C15—C16—H16	119.9
C6—C5—C4	121.1 (4)	C15—C16—C17	120.2 (4)
C6—C5—H5	119.5	C17—C16—H16	119.9
C1—C6—H6	120.1	C16—C17—H17	119.9
C5—C6—C1	119.8 (4)	C18—C17—C16	120.2 (4)
C5—C6—H6	120.1	C18—C17—H17	119.9
N1—C7—N3	128.8 (4)	C17—C18—H18	119.9
N1—C7—C4	114.6 (4)	C17—C18—C19	120.2 (4)
N3—C7—C4	116.5 (4)	C19—C18—H18	119.9
C9—C8—N2	116.9 (4)	C14—C19—H19	119.9
C9—C8—C13	119.7 (4)	C18—C19—C14	120.1 (4)
C13—C8—N2	123.3 (4)	C18—C19—H19	119.9
C8—C9—H9	120.0		
			
Br1—C1—C2—C3	−176.2 (3)	C3—C4—C7—N3	−2.6 (6)
Br1—C1—C6—C5	176.0 (3)	C4—C5—C6—C1	0.0 (6)
N1—N2—C8—C9	−171.6 (3)	C5—C4—C7—N1	−5.7 (5)
N1—N2—C8—C13	8.0 (6)	C5—C4—C7—N3	176.7 (3)
N2—N1—C7—N3	−1.7 (6)	C6—C1—C2—C3	0.8 (6)
N2—N1—C7—C4	−179.0 (3)	C7—N1—N2—C8	176.3 (3)
N2—C8—C9—C10	176.9 (4)	C7—N3—N4—C14	−177.1 (3)
N2—C8—C13—C12	−177.3 (4)	C7—C4—C5—C6	−178.2 (4)
N3—N4—C14—C15	6.4 (6)	C8—C9—C10—C11	1.3 (6)
N3—N4—C14—C19	−175.3 (3)	C9—C8—C13—C12	2.4 (6)
N4—N3—C7—N1	5.4 (6)	C9—C10—C11—C12	0.7 (6)
N4—N3—C7—C4	−177.3 (3)	C10—C11—C12—C13	−1.1 (6)
N4—C14—C15—C16	176.9 (4)	C11—C12—C13—C8	−0.4 (6)
N4—C14—C19—C18	−176.0 (4)	C13—C8—C9—C10	−2.8 (6)
C1—C2—C3—C4	0.3 (7)	C14—C15—C16—C17	0.2 (6)
C2—C1—C6—C5	−1.0 (6)	C15—C14—C19—C18	2.3 (6)
C2—C3—C4—C5	−1.2 (6)	C15—C16—C17—C18	−0.3 (7)
C2—C3—C4—C7	178.1 (4)	C16—C17—C18—C19	1.4 (7)
C3—C4—C5—C6	1.1 (6)	C17—C18—C19—C14	−2.4 (7)
C3—C4—C7—N1	175.0 (4)	C19—C14—C15—C16	−1.2 (6)

### N'-Anilino-4-bromo-N-(phenylimino)benzene-1-carboximidamide (1) Hydrogen-bond geometry (Å, º)

**Table:**

*D*—H···*A*	*D*—H	H···*A*	*D*···*A*	*D*—H···*A*
N2—H2···N4	0.80 (10)	1.93 (10)	2.566 (5)	137 (9)
N4—H4···N1	0.81 (12)	2.40 (11)	2.803 (5)	112 (9)
N4—H4···N2	0.81 (12)	1.86 (12)	2.566 (5)	145 (10)
C19—H19···Br1^i^	0.95	3.05	3.921 (4)	153
C9—H9···Br1^i^	0.95	3.14	4.014 (5)	153

Symmetry code: (i) −*x*+1/2, −*y*+1, *z*+1/2.

### 6-(4-Bromophenyl)-2,4-diphenyl-1,2,3,4-tetrahydro-1,2,4,5-tetrazin-1-yl (2) Crystal data

**Table:**

C_20_H_16_BrN_4_	*D*_x_ = 1.561 Mg m^−^^3^
*M**_r_* = 392.28	Mo *K*α radiation, λ = 0.71073 Å
Orthorhombic, *P**b**c**a*	Cell parameters from 8857 reflections
*a* = 7.0881 (3) Å	θ = 2.7–29.5°
*b* = 21.2183 (11) Å	µ = 2.47 mm^−^^1^
*c* = 22.2028 (9) Å	*T* = 123 K
*V* = 3339.2 (3) Å^3^	Plate, clear green
*Z* = 8	0.15 × 0.12 × 0.06 mm
*F*(000) = 1592	

### 6-(4-Bromophenyl)-2,4-diphenyl-1,2,3,4-tetrahydro-1,2,4,5-tetrazin-1-yl (2) Data collection

**Table:**

STOE IPDS 2T diffractometer	3640 independent reflections
Radiation source: sealed X-ray tube, 12 x 0.4 mm long-fine focus	3397 reflections with *I* > 2σ(*I*)
Plane graphite monochromator	*R*_int_ = 0.128
Detector resolution: 6.67 pixels mm^-1^	θ_max_ = 27.0°, θ_min_ = 2.7°
rotation method scans	*h* = −9→9
Absorption correction: integration (X-RED32; Stoe & Cie, 2009)	*k* = −26→26
*T*_min_ = 0.254, *T*_max_ = 0.620	*l* = −28→28
70879 measured reflections	

### 6-(4-Bromophenyl)-2,4-diphenyl-1,2,3,4-tetrahydro-1,2,4,5-tetrazin-1-yl (2) Refinement

**Table:**

Refinement on *F*^2^	Primary atom site location: structure-invariant direct methods
Least-squares matrix: full	Hydrogen site location: inferred from neighbouring sites
*R*[*F*^2^ > 2σ(*F*^2^)] = 0.029	H-atom parameters constrained
*wR*(*F*^2^) = 0.076	*w* = 1/[σ^2^(*F*_o_^2^) + (0.0366*P*)^2^ + 1.3621*P*] where *P* = (*F*_o_^2^ + 2*F*_c_^2^)/3
*S* = 1.10	(Δ/σ)_max_ = 0.001
3640 reflections	Δρ_max_ = 0.72 e Å^−^^3^
226 parameters	Δρ_min_ = −0.65 e Å^−^^3^
0 restraints	

### 6-(4-Bromophenyl)-2,4-diphenyl-1,2,3,4-tetrahydro-1,2,4,5-tetrazin-1-yl (2) Special details

**Table:**

Geometry. All esds (except the esd in the dihedral angle between two l.s. planes) are estimated using the full covariance matrix. The cell esds are taken into account individually in the estimation of esds in distances, angles and torsion angles; correlations between esds in cell parameters are only used when they are defined by crystal symmetry. An approximate (isotropic) treatment of cell esds is used for estimating esds involving l.s. planes.

### 6-(4-Bromophenyl)-2,4-diphenyl-1,2,3,4-tetrahydro-1,2,4,5-tetrazin-1-yl (2) Fractional atomic coordinates and isotropic or equivalent isotropic displacement parameters (Å^2^)

**Table:**

	*x*	*y*	*z*	*U*_iso_*/*U*_eq_	
Br1	0.68387 (3)	0.06162 (2)	0.45203 (2)	0.02595 (8)	
N1	0.66003 (19)	0.27769 (7)	0.22407 (6)	0.0176 (3)	
N2	0.63370 (19)	0.31170 (6)	0.17288 (6)	0.0177 (3)	
N3	0.5658 (2)	0.22196 (6)	0.11776 (6)	0.0184 (3)	
N4	0.59259 (19)	0.18478 (6)	0.16660 (6)	0.0177 (3)	
C1	0.6208 (2)	0.21623 (7)	0.21812 (7)	0.0168 (3)	
C2	0.5041 (2)	0.28635 (7)	0.12838 (8)	0.0196 (3)	
H2A	0.5096	0.3112	0.0907	0.024*	
H2B	0.3731	0.2870	0.1438	0.024*	
C3	0.6287 (2)	0.17818 (7)	0.27390 (7)	0.0169 (3)	
C4	0.6697 (2)	0.11391 (8)	0.27170 (8)	0.0203 (3)	
H4	0.6898	0.0942	0.2338	0.024*	
C5	0.6815 (2)	0.07845 (8)	0.32404 (8)	0.0219 (3)	
H5	0.7076	0.0346	0.3221	0.026*	
C6	0.6550 (2)	0.10777 (8)	0.37910 (7)	0.0188 (3)	
C7	0.6106 (2)	0.17127 (8)	0.38283 (8)	0.0201 (3)	
H7	0.5912	0.1908	0.4208	0.024*	
C8	0.5950 (2)	0.20578 (7)	0.32989 (7)	0.0182 (3)	
H8	0.5606	0.2490	0.3319	0.022*	
C9	0.7103 (2)	0.37250 (7)	0.16939 (7)	0.0169 (3)	
C10	0.8613 (2)	0.38881 (8)	0.20700 (8)	0.0203 (3)	
H10	0.9078	0.3594	0.2356	0.024*	
C11	0.9424 (3)	0.44801 (8)	0.20224 (8)	0.0230 (4)	
H11	1.0434	0.4593	0.2282	0.028*	
C12	0.8777 (3)	0.49117 (8)	0.15982 (8)	0.0230 (3)	
H12	0.9363	0.5312	0.1560	0.028*	
C13	0.7268 (3)	0.47506 (8)	0.12329 (8)	0.0236 (3)	
H13	0.6818	0.5044	0.0944	0.028*	
C14	0.6404 (2)	0.41659 (8)	0.12830 (8)	0.0204 (3)	
H14	0.5344	0.4066	0.1039	0.024*	
C15	0.5678 (2)	0.19397 (7)	0.06049 (7)	0.0173 (3)	
C16	0.4641 (2)	0.21964 (8)	0.01255 (7)	0.0195 (3)	
H16	0.3903	0.2565	0.0185	0.023*	
C17	0.4698 (2)	0.19094 (8)	−0.04350 (7)	0.0218 (3)	
H17	0.3998	0.2085	−0.0759	0.026*	
C18	0.5761 (3)	0.13688 (8)	−0.05301 (8)	0.0236 (4)	
H18	0.5797	0.1176	−0.0916	0.028*	
C19	0.6775 (2)	0.11138 (8)	−0.00510 (8)	0.0238 (4)	
H19	0.7506	0.0744	−0.0112	0.029*	
C20	0.6733 (2)	0.13926 (8)	0.05142 (8)	0.0202 (3)	
H20	0.7420	0.1212	0.0838	0.024*	

### 6-(4-Bromophenyl)-2,4-diphenyl-1,2,3,4-tetrahydro-1,2,4,5-tetrazin-1-yl (2) Atomic displacement parameters (Å^2^)

**Table:**

	*U*^11^	*U*^22^	*U*^33^	*U*^12^	*U*^13^	*U*^23^
Br1	0.02689 (12)	0.02657 (12)	0.02438 (12)	−0.00268 (6)	−0.00495 (6)	0.00843 (6)
N1	0.0164 (6)	0.0181 (6)	0.0184 (7)	0.0001 (5)	0.0007 (5)	0.0015 (5)
N2	0.0176 (6)	0.0169 (6)	0.0186 (6)	−0.0016 (5)	−0.0021 (5)	0.0006 (5)
N3	0.0206 (7)	0.0166 (6)	0.0180 (6)	0.0007 (5)	−0.0028 (5)	−0.0001 (5)
N4	0.0166 (6)	0.0183 (6)	0.0181 (6)	0.0002 (5)	−0.0011 (5)	0.0017 (5)
C1	0.0105 (7)	0.0190 (7)	0.0207 (8)	0.0002 (6)	0.0004 (6)	−0.0008 (6)
C2	0.0171 (7)	0.0178 (7)	0.0238 (8)	0.0009 (6)	−0.0038 (6)	−0.0009 (6)
C3	0.0106 (7)	0.0190 (7)	0.0211 (8)	−0.0020 (6)	−0.0005 (6)	0.0008 (6)
C4	0.0201 (8)	0.0200 (8)	0.0209 (8)	−0.0002 (6)	−0.0003 (6)	−0.0028 (6)
C5	0.0198 (8)	0.0176 (7)	0.0282 (9)	0.0005 (6)	−0.0013 (6)	0.0011 (7)
C6	0.0148 (7)	0.0219 (8)	0.0197 (8)	−0.0033 (6)	−0.0021 (6)	0.0053 (6)
C7	0.0180 (8)	0.0215 (8)	0.0209 (8)	−0.0017 (6)	0.0008 (6)	−0.0009 (6)
C8	0.0156 (7)	0.0176 (7)	0.0213 (8)	−0.0010 (6)	0.0008 (6)	−0.0002 (6)
C9	0.0156 (7)	0.0150 (7)	0.0202 (8)	0.0003 (5)	0.0031 (6)	−0.0009 (6)
C10	0.0200 (8)	0.0199 (8)	0.0210 (8)	−0.0003 (6)	−0.0020 (6)	0.0014 (6)
C11	0.0211 (8)	0.0223 (8)	0.0256 (9)	−0.0024 (6)	−0.0032 (7)	−0.0015 (6)
C12	0.0240 (8)	0.0166 (7)	0.0284 (9)	−0.0026 (6)	0.0022 (7)	−0.0005 (6)
C13	0.0246 (8)	0.0193 (8)	0.0268 (9)	0.0030 (7)	−0.0019 (7)	0.0019 (7)
C14	0.0189 (8)	0.0191 (8)	0.0231 (8)	0.0002 (6)	−0.0033 (6)	−0.0005 (6)
C15	0.0153 (7)	0.0186 (7)	0.0180 (7)	−0.0044 (6)	−0.0001 (6)	0.0004 (6)
C16	0.0169 (7)	0.0190 (7)	0.0227 (8)	−0.0025 (6)	−0.0022 (6)	0.0011 (6)
C17	0.0197 (8)	0.0255 (8)	0.0203 (8)	−0.0049 (7)	−0.0043 (6)	0.0031 (6)
C18	0.0249 (9)	0.0266 (9)	0.0193 (8)	−0.0057 (7)	0.0017 (6)	−0.0052 (6)
C19	0.0234 (9)	0.0214 (8)	0.0266 (9)	−0.0015 (6)	0.0018 (7)	−0.0035 (7)
C20	0.0185 (8)	0.0195 (8)	0.0227 (8)	−0.0014 (6)	−0.0027 (6)	0.0008 (6)

### 6-(4-Bromophenyl)-2,4-diphenyl-1,2,3,4-tetrahydro-1,2,4,5-tetrazin-1-yl (2) Geometric parameters (Å, º)

**Table:**

Br1—C6	1.9034 (16)	C9—C10	1.401 (2)
N1—N2	1.3592 (19)	C9—C14	1.398 (2)
N1—C1	1.340 (2)	C10—H10	0.9500
N2—C2	1.452 (2)	C10—C11	1.385 (2)
N2—C9	1.402 (2)	C11—H11	0.9500
N3—N4	1.3544 (18)	C11—C12	1.392 (2)
N3—C2	1.454 (2)	C12—H12	0.9500
N3—C15	1.404 (2)	C12—C13	1.385 (3)
N4—C1	1.339 (2)	C13—H13	0.9500
C1—C3	1.479 (2)	C13—C14	1.388 (2)
C2—H2A	0.9900	C14—H14	0.9500
C2—H2B	0.9900	C15—C16	1.404 (2)
C3—C4	1.395 (2)	C15—C20	1.395 (2)
C3—C8	1.395 (2)	C16—H16	0.9500
C4—H4	0.9500	C16—C17	1.386 (2)
C4—C5	1.387 (2)	C17—H17	0.9500
C5—H5	0.9500	C17—C18	1.389 (3)
C5—C6	1.385 (2)	C18—H18	0.9500
C6—C7	1.386 (2)	C18—C19	1.393 (3)
C7—H7	0.9500	C19—H19	0.9500
C7—C8	1.389 (2)	C19—C20	1.388 (2)
C8—H8	0.9500	C20—H20	0.9500
			
C1—N1—N2	113.94 (13)	C14—C9—N2	120.97 (15)
N1—N2—C2	117.33 (13)	C14—C9—C10	119.65 (15)
N1—N2—C9	118.79 (13)	C9—C10—H10	120.1
C9—N2—C2	123.25 (13)	C11—C10—C9	119.71 (15)
N4—N3—C2	117.38 (13)	C11—C10—H10	120.1
N4—N3—C15	118.51 (13)	C10—C11—H11	119.6
C15—N3—C2	123.22 (13)	C10—C11—C12	120.76 (16)
C1—N4—N3	114.49 (13)	C12—C11—H11	119.6
N1—C1—C3	116.15 (14)	C11—C12—H12	120.4
N4—C1—N1	126.88 (15)	C13—C12—C11	119.24 (16)
N4—C1—C3	116.66 (14)	C13—C12—H12	120.4
N2—C2—N3	105.55 (12)	C12—C13—H13	119.5
N2—C2—H2A	110.6	C12—C13—C14	120.96 (16)
N2—C2—H2B	110.6	C14—C13—H13	119.5
N3—C2—H2A	110.6	C9—C14—H14	120.2
N3—C2—H2B	110.6	C13—C14—C9	119.61 (15)
H2A—C2—H2B	108.8	C13—C14—H14	120.2
C4—C3—C1	120.79 (15)	N3—C15—C16	121.15 (14)
C8—C3—C1	120.70 (14)	C20—C15—N3	119.24 (14)
C8—C3—C4	118.51 (15)	C20—C15—C16	119.60 (15)
C3—C4—H4	119.6	C15—C16—H16	120.2
C5—C4—C3	120.90 (16)	C17—C16—C15	119.68 (15)
C5—C4—H4	119.6	C17—C16—H16	120.2
C4—C5—H5	120.4	C16—C17—H17	119.5
C6—C5—C4	119.19 (15)	C16—C17—C18	121.01 (15)
C6—C5—H5	120.4	C18—C17—H17	119.5
C5—C6—Br1	120.35 (13)	C17—C18—H18	120.5
C5—C6—C7	121.36 (15)	C17—C18—C19	119.00 (15)
C7—C6—Br1	118.28 (13)	C19—C18—H18	120.5
C6—C7—H7	120.7	C18—C19—H19	119.5
C6—C7—C8	118.69 (15)	C20—C19—C18	120.90 (16)
C8—C7—H7	120.7	C20—C19—H19	119.5
C3—C8—H8	119.4	C15—C20—H20	120.1
C7—C8—C3	121.26 (14)	C19—C20—C15	119.79 (16)
C7—C8—H8	119.4	C19—C20—H20	120.1
C10—C9—N2	119.37 (14)		

### 6-(4-Bromophenyl)-2,4-diphenyl-1,2,3,4-tetrahydro-1,2,4,5-tetrazin-1-yl (2) Hydrogen-bond geometry (Å, º)

**Table:**

*D*—H···*A*	*D*—H	H···*A*	*D*···*A*	*D*—H···*A*
C10—H10···N1^i^	0.95	2.65	3.519 (2)	153

Symmetry code: (i) *x*+1/2, *y*, −*z*+1/2.

### 5-Anilino-3-(4-bromophenyl)-1-phenyl-1H-1,2,4-triazole (3) Crystal data

**Table:**

C_20_H_15_BrN_4_	*D*_x_ = 1.564 Mg m^−^^3^
*M**_r_* = 391.27	Mo *K*α radiation, λ = 0.71073 Å
Orthorhombic, *P**b**c**a*	Cell parameters from 1652 reflections
*a* = 7.7989 (9) Å	θ = 2.8–23.1°
*b* = 18.971 (3) Å	µ = 2.48 mm^−^^1^
*c* = 22.455 (4) Å	*T* = 100 K
*V* = 3322.4 (8) Å^3^	Plank, clear light yellow
*Z* = 8	0.32 × 0.16 × 0.1 mm
*F*(000) = 1584	

### 5-Anilino-3-(4-bromophenyl)-1-phenyl-1H-1,2,4-triazole (3) Data collection

**Table:**

Bruker X8 Kappa APEXII diffractometer	3999 independent reflections
Radiation source: sealed tube	2708 reflections with *I* > 2σ(*I*)
Graphite monochromator	*R*_int_ = 0.098
Detector resolution: 8 pixels mm^-1^	θ_max_ = 28.0°, θ_min_ = 3.0°
fine slicing ω and φ scans	*h* = −6→10
Absorption correction: multi-scan (SADABS; Bruker, 2015)	*k* = −24→25
*T*_min_ = 0.583, *T*_max_ = 0.746	*l* = −29→29
19450 measured reflections	

### 5-Anilino-3-(4-bromophenyl)-1-phenyl-1H-1,2,4-triazole (3) Refinement

**Table:**

Refinement on *F*^2^	0 restraints
Least-squares matrix: full	Hydrogen site location: mixed
*R*[*F*^2^ > 2σ(*F*^2^)] = 0.056	H atoms treated by a mixture of independent and constrained refinement
*wR*(*F*^2^) = 0.126	*w* = 1/[σ^2^(*F*_o_^2^) + (0.0408*P*)^2^ + 6.8615*P*] where *P* = (*F*_o_^2^ + 2*F*_c_^2^)/3
*S* = 1.05	(Δ/σ)_max_ = 0.001
3999 reflections	Δρ_max_ = 1.46 e Å^−^^3^
229 parameters	Δρ_min_ = −0.84 e Å^−^^3^

### 5-Anilino-3-(4-bromophenyl)-1-phenyl-1H-1,2,4-triazole (3) Special details

**Table:**

Geometry. All esds (except the esd in the dihedral angle between two l.s. planes) are estimated using the full covariance matrix. The cell esds are taken into account individually in the estimation of esds in distances, angles and torsion angles; correlations between esds in cell parameters are only used when they are defined by crystal symmetry. An approximate (isotropic) treatment of cell esds is used for estimating esds involving l.s. planes.

### 5-Anilino-3-(4-bromophenyl)-1-phenyl-1H-1,2,4-triazole (3) Fractional atomic coordinates and isotropic or equivalent isotropic displacement parameters (Å^2^)

**Table:**

	*x*	*y*	*z*	*U*_iso_*/*U*_eq_	
Br1	1.11136 (5)	0.36858 (2)	0.25928 (2)	0.02548 (14)	
N1	0.7607 (4)	0.57251 (19)	0.48352 (14)	0.0201 (7)	
N2	0.6739 (4)	0.58069 (18)	0.53638 (14)	0.0179 (7)	
N3	0.7073 (4)	0.46533 (19)	0.52531 (15)	0.0193 (7)	
N4	0.5536 (4)	0.5068 (2)	0.61156 (16)	0.0249 (8)	
H4	0.487 (6)	0.549 (3)	0.626 (2)	0.030*	
C1	0.7806 (5)	0.5035 (2)	0.48020 (17)	0.0178 (8)	
C2	0.6420 (5)	0.5157 (2)	0.55932 (17)	0.0191 (9)	
C3	0.8667 (4)	0.4696 (2)	0.42895 (17)	0.0183 (8)	
C4	0.8470 (5)	0.3977 (2)	0.41832 (18)	0.0195 (9)	
H4A	0.7837	0.3694	0.4454	0.023*	
C5	0.9206 (5)	0.3673 (2)	0.36779 (18)	0.0216 (8)	
H5	0.9072	0.3184	0.3601	0.026*	
C6	1.0134 (4)	0.4094 (2)	0.32914 (17)	0.0174 (8)	
C7	1.0376 (5)	0.4806 (2)	0.33950 (18)	0.0220 (9)	
H7	1.1034	0.5085	0.3128	0.026*	
C8	0.9637 (5)	0.5105 (2)	0.38984 (18)	0.0203 (9)	
H8	0.9795	0.5593	0.3977	0.024*	
C9	0.6007 (5)	0.6479 (2)	0.55219 (17)	0.0176 (8)	
C10	0.5417 (5)	0.6904 (2)	0.50640 (18)	0.0216 (9)	
H10	0.5527	0.6759	0.4661	0.026*	
C11	0.4660 (5)	0.7548 (2)	0.5203 (2)	0.0266 (10)	
H11	0.4264	0.7849	0.4893	0.032*	
C12	0.4485 (5)	0.7750 (2)	0.5792 (2)	0.0274 (10)	
H12	0.3939	0.8184	0.5886	0.033*	
C13	0.5099 (5)	0.7328 (2)	0.6240 (2)	0.0269 (10)	
H13	0.4985	0.7471	0.6643	0.032*	
C14	0.5883 (5)	0.6694 (2)	0.61057 (18)	0.0219 (9)	
H14	0.6334	0.6407	0.6415	0.026*	
C15	0.5186 (5)	0.4420 (2)	0.64132 (19)	0.0237 (9)	
C16	0.3863 (5)	0.4433 (3)	0.68331 (19)	0.0276 (10)	
H16	0.3253	0.4858	0.6904	0.033*	
C17	0.3441 (6)	0.3832 (3)	0.7144 (2)	0.0313 (11)	
H17	0.2541	0.3846	0.7429	0.038*	
C18	0.4312 (6)	0.3214 (3)	0.7045 (2)	0.0302 (11)	
H18	0.3998	0.2798	0.7253	0.036*	
C19	0.5661 (6)	0.3199 (2)	0.66360 (19)	0.0268 (10)	
H19	0.6285	0.2775	0.6576	0.032*	
C20	0.6096 (5)	0.3798 (2)	0.63171 (18)	0.0249 (9)	
H20	0.7006	0.3785	0.6036	0.030*	

### 5-Anilino-3-(4-bromophenyl)-1-phenyl-1H-1,2,4-triazole (3) Atomic displacement parameters (Å^2^)

**Table:**

	*U*^11^	*U*^22^	*U*^33^	*U*^12^	*U*^13^	*U*^23^
Br1	0.0214 (2)	0.0350 (3)	0.0200 (2)	0.00676 (19)	0.00321 (17)	−0.00357 (19)
N1	0.0195 (16)	0.023 (2)	0.0179 (17)	0.0000 (14)	0.0018 (14)	−0.0011 (14)
N2	0.0199 (16)	0.0151 (19)	0.0186 (17)	0.0001 (13)	0.0037 (13)	−0.0002 (14)
N3	0.0164 (16)	0.019 (2)	0.0223 (18)	−0.0010 (14)	0.0001 (13)	−0.0017 (15)
N4	0.0240 (18)	0.023 (2)	0.0283 (19)	0.0022 (15)	0.0046 (15)	0.0009 (16)
C1	0.0120 (18)	0.024 (2)	0.0180 (19)	−0.0008 (16)	−0.0004 (15)	0.0017 (17)
C2	0.0179 (19)	0.020 (2)	0.019 (2)	0.0022 (16)	−0.0001 (15)	0.0007 (16)
C3	0.0143 (18)	0.022 (2)	0.0182 (19)	0.0018 (16)	−0.0024 (15)	−0.0003 (16)
C4	0.0168 (18)	0.018 (2)	0.023 (2)	0.0009 (16)	0.0009 (15)	0.0015 (17)
C5	0.0208 (19)	0.015 (2)	0.029 (2)	−0.0001 (17)	0.0007 (16)	−0.0008 (18)
C6	0.0104 (17)	0.024 (2)	0.0179 (19)	0.0058 (16)	0.0000 (15)	−0.0016 (16)
C7	0.0167 (18)	0.025 (3)	0.025 (2)	0.0012 (17)	0.0040 (16)	0.0028 (18)
C8	0.0205 (19)	0.015 (2)	0.025 (2)	−0.0013 (17)	−0.0007 (17)	−0.0007 (17)
C9	0.0143 (17)	0.013 (2)	0.026 (2)	−0.0047 (15)	−0.0005 (16)	0.0029 (15)
C10	0.0191 (19)	0.025 (3)	0.021 (2)	−0.0003 (17)	−0.0007 (16)	−0.0001 (17)
C11	0.022 (2)	0.023 (3)	0.035 (3)	−0.0014 (18)	−0.0046 (18)	0.012 (2)
C12	0.024 (2)	0.013 (2)	0.046 (3)	0.0018 (17)	0.0001 (19)	−0.002 (2)
C13	0.027 (2)	0.026 (3)	0.027 (2)	−0.0007 (19)	−0.0026 (18)	−0.0048 (19)
C14	0.021 (2)	0.021 (2)	0.024 (2)	−0.0017 (17)	−0.0023 (17)	0.0037 (17)
C15	0.025 (2)	0.023 (3)	0.023 (2)	−0.0075 (18)	−0.0061 (18)	0.0047 (18)
C16	0.027 (2)	0.029 (3)	0.027 (2)	0.000 (2)	0.0024 (19)	−0.0022 (19)
C17	0.032 (2)	0.037 (3)	0.025 (2)	−0.005 (2)	0.0035 (19)	0.001 (2)
C18	0.038 (3)	0.026 (3)	0.026 (2)	−0.009 (2)	−0.0014 (19)	0.0046 (19)
C19	0.033 (2)	0.017 (2)	0.030 (2)	−0.0013 (18)	−0.0049 (18)	−0.0012 (18)
C20	0.023 (2)	0.029 (3)	0.022 (2)	−0.0040 (19)	−0.0010 (17)	−0.0018 (17)

### 5-Anilino-3-(4-bromophenyl)-1-phenyl-1H-1,2,4-triazole (3) Geometric parameters (Å, º)

**Table:**

Br1—C6	1.909 (4)	C9—C14	1.376 (6)
N1—N2	1.375 (4)	C10—H10	0.9500
N1—C1	1.321 (5)	C10—C11	1.392 (6)
N2—C2	1.358 (5)	C11—H11	0.9500
N2—C9	1.441 (5)	C11—C12	1.384 (6)
N3—C1	1.370 (5)	C12—H12	0.9500
N3—C2	1.326 (5)	C12—C13	1.373 (6)
N4—H4	1.00 (5)	C13—H13	0.9500
N4—C2	1.371 (5)	C13—C14	1.382 (6)
N4—C15	1.426 (6)	C14—H14	0.9500
C1—C3	1.479 (5)	C15—C16	1.398 (6)
C3—C4	1.393 (6)	C15—C20	1.394 (6)
C3—C8	1.395 (6)	C16—H16	0.9500
C4—H4A	0.9500	C16—C17	1.376 (6)
C4—C5	1.397 (6)	C17—H17	0.9500
C5—H5	0.9500	C17—C18	1.374 (7)
C5—C6	1.384 (6)	C18—H18	0.9500
C6—C7	1.384 (6)	C18—C19	1.396 (6)
C7—H7	0.9500	C19—H19	0.9500
C7—C8	1.390 (6)	C19—C20	1.385 (6)
C8—H8	0.9500	C20—H20	0.9500
C9—C10	1.386 (5)		
			
C1—N1—N2	102.6 (3)	C14—C9—C10	120.7 (4)
N1—N2—C9	120.5 (3)	C9—C10—H10	120.5
C2—N2—N1	108.4 (3)	C9—C10—C11	119.1 (4)
C2—N2—C9	129.5 (3)	C11—C10—H10	120.5
C2—N3—C1	101.8 (3)	C10—C11—H11	120.0
C2—N4—H4	116 (3)	C12—C11—C10	119.9 (4)
C2—N4—C15	127.1 (4)	C12—C11—H11	120.0
C15—N4—H4	116 (3)	C11—C12—H12	119.9
N1—C1—N3	115.7 (4)	C13—C12—C11	120.3 (4)
N1—C1—C3	121.9 (4)	C13—C12—H12	119.9
N3—C1—C3	122.3 (4)	C12—C13—H13	119.9
N2—C2—N4	121.9 (4)	C12—C13—C14	120.2 (4)
N3—C2—N2	111.4 (3)	C14—C13—H13	119.9
N3—C2—N4	126.7 (4)	C9—C14—C13	119.8 (4)
C4—C3—C1	120.6 (4)	C9—C14—H14	120.1
C4—C3—C8	119.7 (4)	C13—C14—H14	120.1
C8—C3—C1	119.6 (4)	C16—C15—N4	116.2 (4)
C3—C4—H4A	120.0	C20—C15—N4	124.1 (4)
C3—C4—C5	119.9 (4)	C20—C15—C16	119.7 (4)
C5—C4—H4A	120.0	C15—C16—H16	119.9
C4—C5—H5	120.5	C17—C16—C15	120.3 (4)
C6—C5—C4	119.0 (4)	C17—C16—H16	119.9
C6—C5—H5	120.5	C16—C17—H17	119.8
C5—C6—Br1	119.4 (3)	C18—C17—C16	120.4 (4)
C5—C6—C7	122.0 (4)	C18—C17—H17	119.8
C7—C6—Br1	118.7 (3)	C17—C18—H18	120.1
C6—C7—H7	120.7	C17—C18—C19	119.7 (4)
C6—C7—C8	118.6 (4)	C19—C18—H18	120.1
C8—C7—H7	120.7	C18—C19—H19	119.7
C3—C8—H8	119.7	C20—C19—C18	120.5 (4)
C7—C8—C3	120.7 (4)	C20—C19—H19	119.7
C7—C8—H8	119.7	C15—C20—H20	120.4
C10—C9—N2	117.7 (4)	C19—C20—C15	119.3 (4)
C14—C9—N2	121.7 (4)	C19—C20—H20	120.4

### 5-Anilino-3-(4-bromophenyl)-1-phenyl-1H-1,2,4-triazole (3) Hydrogen-bond geometry (Å, º)

**Table:**

*D*—H···*A*	*D*—H	H···*A*	*D*···*A*	*D*—H···*A*
C14—H14···Br1^i^	0.95	2.99	3.814 (4)	146 (1)
C10—H10···C20^ii^	0.95	2.75	3.575 (5)	146 (1)
C17—H17···C19^iii^	0.95	2.84	3.694 (6)	150 (1)

Symmetry codes: (i) −*x*+2, −*y*+1, −*z*+1; (ii) −*x*+1, −*y*+1, −*z*+1; (iii) *x*−1/2, *y*, −*z*+3/2.
